# Applications of Fluorescence Technology for Rapid Identification of Marine Plastic Pollution

**DOI:** 10.3390/polym17121679

**Published:** 2025-06-17

**Authors:** Haoyu Zhang, Yanjun Li, Lixin Zhu, Xindi Song, Changbin Ren, Buyu Guo, Yanzhen Gu

**Affiliations:** 1 Ocean College, Zhejiang University, Zhoushan 316021, China; 22334099@zju.edu.cn (H.Z.); 22334115@zju.edu.cn (X.S.); 2 Hainan Institute, Zhejiang University, Sanya 572025, China; renchangbin6936@126.com (C.R.); guobuyuwork@163.com (B.G.); 3 Hainan Observation and Research Station of Ecological Environment and Fishery Resource in Yazhou Bay, Sanya 572025, China; 4 State Key Laboratory of Estuarine and Coastal Research, East China Normal University, Shanghai 200241, China; lixinzhu0305@hotmail.com; 5 Donghai Laboratory, Zhoushan 316021, China

**Keywords:** fluorescence technology, fluorescence lifetime, fluorescent dyes, microplastics, macrolitter, plastic pollution, identification

## Abstract

As global plastic production increases, the problem of marine plastic pollution is becoming increasingly critical, and the development of effective identification technologies is particularly urgent as plastic debris not only poses a threat to aquatic ecosystems but also has a significant impact on human health. This paper presents the criteria for evaluating fluorescence technology and its mechanism for plastic identification, with an emphasis on its potential for the rapid detection of marine plastic pollution. By analyzing variations in the fluorescence lifetimes and intensities of plastics, different types of plastics can be effectively distinguished. In addition, this paper reviews the detection of microplastics using different fluorescent dyes and explores the fluorescence lifetime identification method. This paper also demonstrates the effectiveness of fluorescence techniques for macroplastic identification, highlighting how fluorescence lifetimes and decay rates change in various weathering environments. Monitoring these changes offers a foundation for establishing weathering models, aiding in understanding the transformation of macrolitter into microplastics. Future research should investigate the autofluorescence properties of different plastics further and focus on developing detection methods and instruments for various environments. This will improve the identification of plastic waste in complex environments. In conclusion, fluorescence technology shows great promise in plastic identification and is expected to provide substantial support for recycling plastic waste products and mitigating plastic pollution.

## 1. Introduction

Global plastic production has escalated dramatically, from 1.5 million tons in the 1950s to nearly 370 million tons by 2019 [1], with an estimated 4.8 to 12.7 million tons of plastic waste entering the oceans each year [2]. Plastic debris is now pervasive across oceanic environments, including coastal areas, seabeds, and the sea surface [3]. These plastics, predominantly composed of packaging materials and household items [4], are lightweight, durable, and water-insoluble, characteristics that contribute to their resistance to degradation, even after prolonged exposure and aging processes. This group of plastics, known as macrolitter, includes plastic litter items between 25 and 1000 mm [5]. Upon entering marine environments, macrolitter gradually fragments into smaller particles categorized by size: microplastics (MPs), typically ranging from 1 to 5000 μm, and nanoplastics (NPs), which are even smaller [6].

In 2021, Morales-Caselles et al. conducted a comprehensive global study on the distribution of litter in oceans and rivers [7], revealing the widespread impact of plastic waste as the primary source of pollution across marine environments, and microplastics were found from coastal areas to the deepest trenches, such as the Mariana Trench [8]. Fisheries-related debris and plastic bottles were identified as the most pervasive types of plastic pollution. This issue is particularly acute along coastlines, where plastic waste accumulates extensively, and significant quantities settle on the seabed.

Macrolitter and its fragments, whether covering the seabed or floating on the sea surface, can obstruct coral reefs from accessing light and nutrients [9], thereby altering local ecosystems. These plastics may also be ingested by marine organisms, causing blockage in their digestive tracts, malnutrition, and even death [10]. Additionally, plastics can absorb persistent organic pollutants from the marine environment [11]. As macrolitter degrades into microplastics (MNPs), they can be consumed by phytoplankton, thereby entering the food chain. Through the biomagnification effect, toxic substances from microplastics accumulate progressively across trophic levels [12]. Furthermore, MNPs can penetrate biological cells and tissues [13], and their absorption by marine organisms has been shown to cause adverse effects such as reduced feeding rate, diminished body weight, lowered metabolic rate [14], and increased mortality [15]. MNPs are resistant to decomposition, and the consumption of marine organisms containing MNPs may pose a significant risk to human health [16]. Consequently, it is imperative to implement regulatory measures targeting plastic production and recycling processes to mitigate the adverse effects of plastic waste on marine ecosystems.

At the production level, plastics constitute a substantial portion of global material output and are integral to daily life across all regions in 2019, as reported by the Organisation for Economic Cooperation and Development [17]. Despite advancements in plastic production and recycling technologies, a fully efficient recycling infrastructure has yet to be developed [2]. While macrolitter accounts for a significant share of marine litter [18], much of the current research on marine plastics focuses on the identification and management of microplastics [19]. As a result, macrolitter remains inadequately characterized, and the absence of effective identification technologies has resulted in a mere 9% recycling rate for plastics [20], contributing to severe and persistent environmental pollution. To address the challenges posed by marine plastics, it is essential to focus on both detecting and distinguishing environmental MNPs and identifying larger plastics on the water’s surface, within the water column, and on the bottom. Developing effective strategies to locate and salvage this macrolitter is crucial for reducing oceanic pollution and mitigating the associated risks to marine life and human health.

Current in situ sampling methods for analyzing marine plastics encompass several approaches:

Trawl Sampling: This technique utilizes various trawl types, including surface trawls [21], manta nets, mesopelagic trawls [22], and bongo nets. These trawls are commonly deployed from both surface waters and mesopelagic layers. Towed sampling primarily captures surface plastics and larger plastic fragments within the mesopelagic zone [23]. However, trawls are also used for benthic sampling and are primarily used to collect samples near the seafloor, including sediments and benthic organisms [24].

Sediment Sampling: This approach targets both surface and benthic sediments, with surface samples often collected from beaches and estuaries using stainless steel spoons and spatulas at low tide [25]. Box corers are also employed to sample surface layers [26], while seabed sediments are sampled using sediment cores [27] and seabed trawls targeting seafloor debris [28].

Despite these approaches, the marine environment’s complexity presents substantial challenges in monitoring plastic distribution. Factors such as ocean currents [29], wind speed, weather patterns [30], and underwater topography [31] result in spatial and temporal variability of plastic pollutants, complicating the attainment of consistent and reliable data. Furthermore, marine organisms exposed to plastic pollution face additional stressors, including heavy metals, organic pollutants, and climate change impacts [32]. Thus, evaluating plastic pollution in isolation may yield limited insight into its broader ecological effects.

The urgent need to develop efficient plastic identification and recycling technologies has spurred advances in detection methods for micro- and nanoparticles of plastics. Current detection approaches include destructive techniques such as Thermal Desorption Gas Chromatography–Mass Spectrometry (TDS-GC/MS) [33], Pyrolysis Gas Chromatography–Mass Spectrometry (Py-GC/MS) [34], and non-destructive methods like Fourier Transform Infrared (FTIR) and Raman spectroscopy [35,36]. TDS-GC-MS exhibits high detection sensitivity for compounds at the part per billion (ppb) level and requires simple sample preparation. However, its applicability is restricted to volatile or semivolatile compounds, and samples are susceptible to cross-contamination [37]. Py-GC/MS offers comprehensive chemical analysis but is hindered by lengthy processing times, complex analytical procedures, and challenges in differentiating complex mixtures [38]. FTIR is suitable for the analysis of polar molecules and provides rapid detection. Nevertheless, it suffers from low spatial resolution and significant interference from water, necessitating specialized sample treatment [39]. Raman spectroscopy is suitable for analyzing nonpolar and symmetric molecules, with minimal water interference and no need for extensive sample preparation. However, it is characterized by weak signal intensity and requires uniform samples [40].

Fluorescence technology, by contrast, has emerged as a promising method for rapid plastic identification in environmental studies. Compared with FTIR and Raman spectroscopy, fluorescence methods offer significantly enhanced detection speed and sensitivity. For instance, fluorescence detection can be up to 1000 times faster than traditional absorption spectroscopy [41], with nanosecond-scale time resolution [42] that enables high-throughput analysis and provides high specificity, clear spectral data, and simple pre-processing [43]. Since different types of plastics exhibit distinct fluorescence lifetimes, measuring and analyzing these lifetimes can significantly improve the accuracy of plastic identification. Moreover, fluorescence lifetime measurements allow for the differentiation of plastic types with high specificity, often yielding identification accuracies above 90% when coupled with machine learning algorithms. [44] In contrast, FTIR and Raman techniques, though valuable for chemical structure elucidation, typically require longer acquisition times (several seconds to minutes per sample) and may suffer from signal overlap in complex matrices [45,46]. The fluorescence approach, particularly when using time-resolved techniques, offers better tolerance to environmental noise and minimal sample preparation. In fluorescence technology, key components include fluorophores and fluorescence. Fluorophores are compounds that absorb and re-emit light at specific wavelengths and contain multiple aromatic rings or conjugated double bonds [47]. Fluorescence is a remarkable photoluminescence phenomenon generated when a fluorophore absorbs radiant energy at a defined wavelength [42]. Plastics contain polycyclic aromatic compounds, ketone groups, and other functional groups capable of producing fluorescent effects [48]. Leveraging this property, researchers have employed two and three-dimensional fluorescence spectroscopy techniques to stain microplastics with fluorescent dyes or induce autofluorescence. This approach facilitates the differentiation and identification of various plastic types [49].

Despite these benefits, two- and three-dimensional fluorescence spectroscopy faces challenges when additives in plastics produce similar spectral peaks, hindering clear differentiation [50]. To address this, time-resolved spectroscopy utilizing fluorescence lifetime imaging microscopy leverages the unique fluorescence lifetimes and quantum efficiencies of each plastic type, allowing more specific plastic identification. To harness the full potential of fluorescence-based plastic identification, plastics can be categorized by detection technique: those identified directly by fluorescence spectroscopy, those combined with fluorescent dyes, and those differentiated by fluorescence lifetime through autofluorescence excitation. These methods provide a holistic approach to plastic identification. Additionally, this review discusses the factors influencing the feasibility of identifying large plastic fragments using fluorescence techniques. Notably, fluorescence lifetime can serve as an indicator of the weathering degree in ocean-exposed plastics, revealing insights into the degradation of marcoplastics.

This review provides a comprehensive overview of the following:

(1) The foundational principles of fluorescence technology.
(2) Fluorescence spectra used for plastics characterization, including characteristic spectral ranges for various types of plastics.
(3) Fluorescent dyes and staining methods for microplastic detection.
(4) Fluorescence lifetimes of various types of plastics under different backgrounds.
(5) The feasibility of using fluorescence technology for identifying large plastic items and influencing factors.

In Section 2, we discuss the principles of fluorescence technology, fluorophore properties, and factors affecting fluorescence lifetime. We highlight its unique role in plastic identification and present the development of different fluorescence spectra, including spectral characteristics and experimental protocols for each plastic type, along with a theoretical assessment of underwater fluorescence plastic identification. Section 3 focuses on fluorescent dyes used in macroplastic identification, detailing the advantages and disadvantages of various dyes and their applicability to specific plastic types. We summarize the fluorescence lifetimes of microplastics and explore the feasibility of fluorescence methods for identifying large plastics, demonstrating the relationship between the fluorescence lifetime of plastics, the structure of the plastic, and the degree of ocean weathering. Section 4 presents the future of fluorescence-based plastic identification, noting the slow progress in autofluorescence technology. Establishing a comprehensive fluorescence database for plastic would significantly accelerate advancements in plastic identification and classification methods.

## 2. Fluorescent Technology and Fluorescent Substances

### 2.1. Principles and Types of Fluorescence Spectroscopy

**Figure 1:**
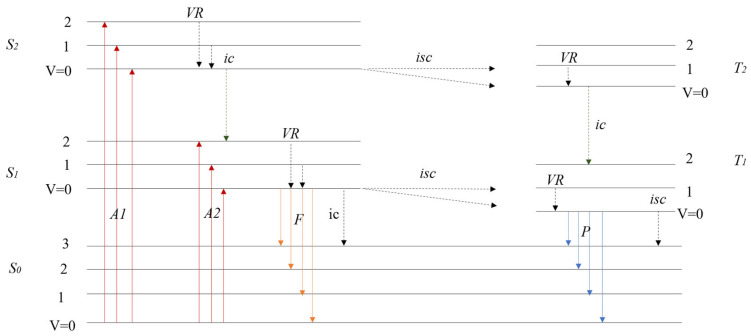
Intramolecular electronically excited decay processes.

Fluorescence spectroscopy is a widely used analytical technique for investigating the photophysical properties of substances [42]. When a fluorescent molecule absorbs light at a specific wavelength, electrons are excited to a higher energy state. These electrons then return to the ground state by emitting fluorescence photons, a process illustrated in Figure 1 [51]. The quantum yield, defined as the ratio of emitted to absorbed photons, quantifies the efficiency of fluorescence emission [52].

Due to non-radiative relaxation processes following excitation, the emitted fluorescence typically has a longer wavelength than the absorbed light—a phenomenon known as the Stokes shift. This shift primarily arises from vibrational relaxation within the excited state and solvent reorganization around the excited molecule prior to photon emission [51]. Fluorescence spectra are produced by measuring the intensity of both excitation light and the emitted fluorescence [51]. Subsequent qualitative and quantitative analysis can be performed based on parameters such as fluorescence wavelength, intensity, polarization, and lifetime, applying principles like Moseley’s law [53] and Sherman’s equation [54]. Functional groups within plastics affect their fluorescence properties. For instance, electron-donating groups like -OH, -NH2, and -OCH3 enhance fluorescence, whereas electron-accepting groups such as -COOH and -N=N- reduce it [55]. Additionally, certain fluorescent groups, including tryptophan and tyrosine, exhibit lower-than-expected quantum yields [56], and elements such as halogens, oxygen, and acrylamide also diminish fluorescence. These variations in fluorescence spectra across plastic types underpin the theoretical framework for plastic identification via fluorescence spectroscopy.

In Figure 1, *VR* denotes vibrational relaxation, *ic* represents internal transformations, and *isc* stands for intersystem crossing. $S_{0}$ is the ground singlet state and the excited singlet states are labeled as $S_{1}$ and $S_{2}$. Similarly, the excited triplet states are denoted as $T_{1}$ and $T_{2}$. The absorption processes are indicated by *A1* and *A2*, fluorescence is abbreviated as *F*, and phosphorescence is represented by *P*.

According to Kasha’s Rule, *F* and *P* almost exclusively occur from the lowest excited state of a given multiplicity—that is, from $S_{1}$ and *T*_1_ (for phosphorescence), regardless of whether higher excited states like $S_{2}$ or $T_{2}$ were initially populated. In the diagram, this is reflected by the fact that emission occurs only from $S_{1}$ and $T_{1}$ after rapid internal *ic* or *isc* from higher states like $S_{2}$ or $T_{2}$.

Additionally, Vavilov’s Rule states that the quantum yield of fluorescence is generally independent of the excitation wavelength. This implies that whether the molecule absorbs light via *A1* or *A2*, the efficiency of fluorescence from $S_{1}$ remains largely unchanged—as long as *ic* efficiently funnels excited populations to $S_{1}$ before emission.

The well-known relationship between the observed fluorescence lifetime ($\tau_{obs}$), the radiative lifetime ($\tau_{r}$), and the fluorescence quantum yield ($\phi_{f}$) is given by the following equation:

(1) $$\phi_{f}=\frac{\tau_{obs}}{\tau_{r}}$$

This equation expresses that the quantum yield of fluorescence is the ratio of the observed decay time (which includes both radiative and non-radiative processes) to the intrinsic radiative lifetime. A higher quantum yield indicates a longer observed lifetime relative to the radiative lifetime, meaning non-radiative processes are less competitive.

Fluorescence spectroscopy, which captures luminescent emissions primarily within the visible spectrum, includes several core techniques: three-dimensional fluorescence spectroscopy (Excitation–Emission Matrix, EEM), two-dimensional fluorescence spectroscopy, time-resolved fluorescence spectroscopy, synchronized fluorescence spectroscopy (SFS), fully synchronized fluorescence spectroscopy (TSFS), and X-ray fluorescence spectroscopy.

Three-dimensional fluorescence spectroscopy, known as Excitation–Emission Matrix (EEM) spectroscopy [57], captures a 3D spectrum encompassing fluorescence wavelength (x), excitation wavelength (y), and fluorescence intensity (z) by employing multiple excitation wavelengths [58]. EEM allows visualization of characteristic peaks and intensity variations for all fluorophores within a sample mixture [59]. This technique has been widely used to monitor dissolved organic matter (DOM) in drinking water sources and treatment plants [60].

Two-dimensional fluorescence spectroscopy encompasses both fluorescence emission and excitation spectra. In fluorescence emission spectroscopy, the incident wavelength is kept constant to observe fluorescence intensity variations across fluorescence wavelengths, with the emission spectrum of a fluorophore mirroring its absorption spectrum [61]. Fluorescence excitation spectroscopy, on the other hand, maintains a fixed fluorescence wavelength and measures intensity across excitation wavelengths, reflecting the electron distribution of a molecule in its ground state [61]. Emission and excitation spectra generally appear as near-mirror images [55]. When optimal excitation wavelengths are defined, emission spectral data can be captured. This distinction between emission and excitation wavelengths enables fluorescent objects to appear significantly brighter than the background in certain spectral regions [62]. Two-dimensional fluorescence has been employed in detecting polycyclic aromatic hydrocarbons (PAHs) in environmental samples such as wastewater and sediment [63]. Fluorescence spectroscopy is promising for underwater applications, as light absorption underwater varies with wavelength [64].

Time-resolved Fluorescence Spectroscopy (TRF) monitors rapid fluorescence changes, on the order of picoseconds, following exposure to ultraviolet, visible, or near-infrared light [65]. TRF characterizes fluorescence lifetimes, aiding in the differentiation of plastic types. For example, Monteleone [66] demonstrated that by analyzing intensity-weighted and amplitude-weighted lifetime values, TRF enabled accurate differentiation of 94.55% of tested micro- and nanoplastics, with or without thermal treatment, highlighting its potential in plastic classification.

In Synchronous Fluorescence Spectroscopy (SFS), excitation and emission wavelengths are adjusted synchronously, maintaining a constant wavelength difference while recording the emission spectrum [67]. Full-Synchronous Fluorescence Spectroscopy (TSFS) extends this technique by generating a contour plot of synchronized spectra across varying offsets, providing detailed data for enhanced sample identification and quantitative analysis. Plastics, particularly those subjected to environmental degradation or combustion, can release polycyclic aromatic hydrocarbons (PAHs), which are persistent and toxic pollutants [68]. SFS has been demonstrated as an effective tool for detecting PAH metabolites, serving as indirect markers of plastic-related contamination. For instance, 1-hydroxypyrene, a primary metabolite of pyrene—a typical PAH associated with plastic pollution—was successfully quantified in marine polychaetes using SFS, with results comparable to those obtained by HPLC-UV methods [69]. This application highlights the potential of SFS for identifying plastic-derived contaminants in environmental and biological samples.

X-ray fluorescence (XRF) spectrometry is a non-destructive atomic analysis method suitable for both qualitative and quantitative analysis of solid samples, including macrolitter and microplastics. XRF measures characteristic fluorescent X-rays emitted from a material after it is bombarded with high-energy X-rays or gamma rays [70]. F Bezati et al. [71] demonstrated the versatility of XRF in identifying tracers in polypropylene, identifying 5 of 7 markers at 1000 ppm concentration with only 1 min exposure, thereby enhancing classification efficiency and improving purity in plastic separation. XRF also addresses the challenge of identifying dark-colored plastics. In 2017, Turner et al. successfully applied portable XRF for in situ elemental characterization of marine microplastics, confirming its feasibility for rapid, accurate in situ analysis of heavy metals and other elements in microplastics [72]. SA Abubaker et al. [73] utilized X-ray diffraction and XRF techniques to differentiate plastic types, High-density polyethylene (HDPE), polyvinyl chloride (PVC), polyethylene terephthalate (PET), and polypropylene (PP), demonstrating XRF’s efficacy in non-destructive waste analysis, thus promoting its application for environmental protection.

These fluorescence-based techniques offer significant promise for plastic identification, with each method providing unique insights that contribute to environmental monitoring and plastic waste management.

### 2.2. Comparison of Fluorescence-Based Imaging Technique

Recent advances in fluorescence-based imaging have significantly expanded the methodological toolkit for plastic identification and characterization in environmental samples, enabling researchers to address diverse analytical challenges [74]. Each imaging modality offers distinct advantages in terms of spatial resolution, detection sensitivity, and throughput, yet also presents inherent limitations that must be carefully considered when analyzing complex environmental matrices [75]. Table 1 provides a comparative summary of key fluorescence imaging techniques employed in microplastic research, systematically evaluating their analytical performance and practical constraints based on recent methodological developments.

As summarized in Table 1, each fluorescence-based imaging technique offers distinct trade-offs in resolution, detection sensitivity, and throughput. CLSM provides superior spatial resolution and 3D imaging capabilities but suffers from low throughput and photobleaching [74,76]. FLIM allows lifetime-based discrimination, enabling quantitative analysis of plastic degradation states, though acquisition time remains a challenge [75]. Widefield microscopy, while fast and accessible, lacks optical sectioning [76]. Imaging flow cytometry offers a high-throughput analysis suitable for population-level plastic detection, albeit with reduced resolution [78]. Two-photon microscopy achieves deep penetration with minimal photodamage, making it suitable for live specimen imaging [80]. These complementary strengths highlight the importance of method selection based on specific analytical goals.

### 2.3. Evaluation Metrics for Fluorescence Spectroscopy

Fluorescence spectroscopy has diverse applications, with fluorescence intensity and peaks serving as primary evaluation metrics. Fluorescence intensity indicates the strength of the emitted light, typically increasing with sample concentration in a controlled laboratory. The fluorescence intensity can be expressed as follows [82]:

(2) $$I_{F}=k\cdot I_{o}\cdot \phi \cdot \left(\varepsilon \cdot b\cdot C\right)$$

where *k* is the instrumental error, $I_{o}$ is the intensity of the incident light, Φ is the quantum yield, ε is the molar extinction coefficient, *b* is the sum of the path lengths, and *C* is the solution molecular concentration.

Fluorescence intensity is linearly correlated with the concentration of pure compounds [83]. However, variables such as solution conditions, temperature, pH, and the presence of other substances can significantly affect intensity. The reduction in fluorescence intensity, known as quenching, is induced by quenching [84]—substances such as oxygen ions [85], acrylamide ions [86], and iodide and cesium ions [87].

The fluorescence peaks—the positions of spectral peaks—reflect the intrinsic properties of fluorescent molecules and are essential to fluorescence spectroscopy. Most peaks arise from molecular absorption and emission, while some result from light scattering phenomena, primarily Rayleigh scattering and Raman scattering [88]. Rayleigh scattering occurs at the excitation wavelength, with scattered photons retaining the same energy as the excited photon. To avoid Rayleigh interference, emission spectra are typically recorded beyond the excitation wavelength [89].

In contrast, Raman scattering appears at wavelengths longer than the excitation wavelength, as scattered photons possess lower energy than excitation photons, resulting in a Raman peak of lower intensity compared to the Rayleigh peak [90]. Raman spectra can complicate fluorescence emission spectra and are typically subtracted to isolate the true fluorescence signal [91]. In aqueous media, the primary Raman peak generally originates from -OH bond vibrations, with its position dependent on the excitation wavelength. The Raman scattering wavelength can be calculated using the following equation:

(3) $$\frac{1}{\lambda_{R}}=\frac{1}{\lambda_{e}}-\bar{v}$$

where $\lambda_{R}$ is the Raman scattering wavelength, $\lambda_{e}$ is the excitation wavelength, and $\bar{v}$ is the Raman shift, which is approximately 3400 to 3600 cm^−1^ for water.

There are two options available to minimize Raman interference. The first is reducing the excitation wavelength, shifting the Raman peak to a lower wavelength and away from the fluorescence wavelength of interest [92]. This method’s effectiveness depends on the fluorophore’s absorption profile and the Stokes shift. For molecules with narrow absorption ranges and small Stokes shifts, such as fluorescein—which absorbs minimally below 400 nm—it is impractical to prevent spectral overlap [93]. The second method involves measuring the fluorophore’s emission spectrum in solution, followed by the spectrum of the solvent alone. By subtracting the solvent spectrum from the solution spectrum, the resulting spectrum of the fluorophore is free from Raman interference. This technique enables the subtraction of solvent-induced background signals, such as Raman scattering and weak autofluorescence, thereby isolating the true emission spectrum of the fluorophore or plastic. This approach improves the specificity of underwater fluorescence-based plastic identification.

### 2.4. Characterization of Fluorophores

Fluorescence lifetime ($\tau_{F}$) and quantum yield (Φ) are critical properties of fluorophores that provide insight into their photophysical behavior. The fluorescence lifetime, often referred to as the average or mean fluorescence lifetime, is defined as the time-weighted average that a fluorophore spends in the excited state before returning to the ground state via photon emission. The fluorescence lifetime represents the average time a molecule remains in its excited state before returning to its ground state, also known as the mean jump time. This parameter is typically measured in nanoseconds [94]. In experimental applications, the fluorescence lifetime decay curve can be approximated as the convolution of the Gaussian system’s response function (width = $\tau_{F}$) and the decay exponent, with the temporal resolution of the photodetector acting as a limiting factor [95]:

(4) $$F\left(t\right)=F_{0}\cdot \frac{1}{2}\exp\left(\frac{\tau_{F}^{2}}{2\tau^{2}}-\frac{t-t_{0}}{\tau }\right)erfc\left(\frac{\tau_{F}^{2}-\tau \left(t-t_{0}\right)}{2\tau \tau_{F}}\right)$$

where $F_{0}$ is the fluorescence intensity immediately after excitation (t = 0), τ is the fluorescence lifetime, t is the point in time when the fluorescent signal is detected, $t_{0}$ is the reference time point of the excitation light pulse.

This function is commonly used in time-resolved fluorescence lifetime analysis to account for the finite width of the excitation pulse. The fluorescence lifetime τ is extracted by fitting this function to experimental decay data via nonlinear least squares or maximum likelihood estimation. Such convolution-based approaches are standard in time-correlated single photon counting and frequency-domain fluorescence lifetime imaging microscopy, as described in Becker [77] and Lakowicz [42]. The use of the complementary error function arises from the analytical convolution of the Gaussian IRF with the exponential decay profile, enabling accurate modeling of the observed fluorescence signal.

Fluorescence lifetime measurement requires specialized instrumentation to capture the fluorescence decay occurring on the nanosecond timescale. Most commercial systems utilize Time-Correlated Single-Photon Counting (TCSPC), which measures the time difference between a single-photon signal detection by a photodetector and the laser pulse initiating the excitation. Fluorescence lifetime imaging (FLIM), integrated with scanning microscopy, enables lifetime measurements on a per-pixel basis. FLIM is often used in conjunction with TCSPC systems due to the employment of pulsed laser excitation. Wide-field illumination and imaging sensors enable faster sampling, particularly for non-light-scattering tissues. Based on this principle, Wei, L et al. developed a time-resolved fluorescence lifetime imaging system using time-gated Complementary Metal–Oxide–Semiconductor (CMOS) sensors capable of measuring fluorescence lifetime in wide-field configurations with high temporal resolution [96]. These CMOS sensors operate based on time-gated detection, wherein the fluorescence decay is sampled at multiple time intervals following a short excitation pulse. The sensor’s integrated timing circuitry allows precise synchronization with the excitation source, enabling pixel-wise lifetime extraction across the entire field of view. Compared to conventional TCSPC systems, CMOS-based FLIM platforms offer higher throughput, lower cost, and are more readily integrated into portable or in vivo imaging devices [97,98]. This approach has demonstrated successful lifetime measurements in live-cell and tissue imaging [98] and holds potential for real-time analysis of fluorescence-labeled plastic particles in environmental samples.

Beyond imaging applications, fluorescence lifetime serves as a sensitive probe of the local physicochemical environment. It is influenced by environmental factors such as pH [99] and temperature, making it a powerful readout for both biological sensing and material characterization. As demonstrated in Table 2, plastics subjected to heat treatment for 12 h exhibit enhanced fluorescence intensity, which persists even after cooling to ambient temperature. This enhancement is likely attributed to a reduction in fluorescence lifetime or an increase in photon yield, both of which contribute to more efficient radiative emission [100]. For single-photon excitation of plastics, shorter wavelengths in the ultraviolet (UV) range prove more effective, as plastic materials exhibit higher light absorption but lower penetration depths. Common excitation wavelengths used for plastic materials include 400 nm, 470 nm, and 405 nm. It is important to consider the instrumental specifications, as the choice of excitation wavelength and detection range depends on the specific equipment and spectral detector applied.

The quantum yield of a fluorophore quantifies its efficiency in emitting photons relative to the number of photons absorbed. It can be expressed as follows:

(5) $$\Phi =\frac{number\;of\;emitted\;photons}{number\;of\;absorbed\;photons}=\frac{k_{rad}}{k_{rad}+k_{nr}}=k_{rad}\tau$$

where $k_{rad}$ is the radiative (fluorescent) decay rate and $k_{nr}$ is the non-radiative decay rate, τ is the fluorescence lifetime.

A high quantum yield is indicative of a fluorophore’s luminescence, achieved when the radiative (fluorescence) decay rate significantly exceeds the non-radiative decay rate. This efficiency is related to the fluorescence lifetime of the fluorophore, with a linear correlation between quantum yield and lifetime [101]. The quantum yield is also sensitive to environmental factors, such as pH, temperature, and solvent properties, making it a valuable indicator of ecological conditions. Extensive research on fluorophores has resulted in the development of comprehensive databases containing optical parameters for standard organic fluorophores. These databases include critical parameters for maximum absorption and emission wavelengths, bandwidths, extinction coefficients, photoluminescence quantum yields, and fluorescence lifetimes [102]. These resources are indispensable for selecting control parameters and ensuring the consistency and reliability of fluorescence-based measurements.

## 3. Plastic Identification by Fluorescence Technology

**Figure 2:**
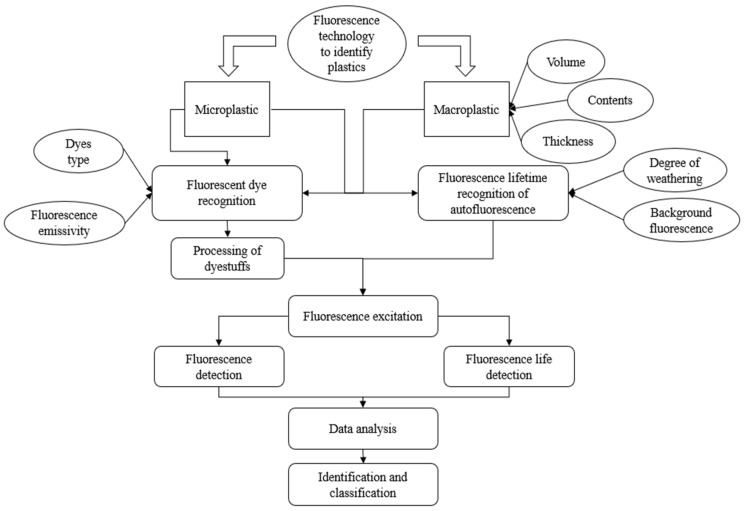
Flowchart and influencing factors for identifying plastics by fluorescence technology.

Plastic identification using fluorescence technology can be categorized into two primary approaches: identification through fluorescent dye labeling and identification via plastic autofluorescence. Figure 2 illustrates the process and key factors influencing plastic identification. The fundamental distinction between these two detection methods lies in the way fluorescence is excited, which affects the key detection parameters, including fluorescence intensity, lifetime, background noise, and other critical detection factors.

This chapter presents these two distinct techniques in detail, addressing their respective mechanisms and applications. Additionally, due to the differences in physical properties between microplastics and macrolitter, the potential for macrolitter identification using fluorescence will be explored, along with an evaluation of existing identification techniques. The goal is to identify commonalities between the identification processes for both macrolitter and microplastics. The plastics discussed in this chapter include HDPE, LDPE, PP, PS, PVC, PA, PU, PC, and PET.

### 3.1. Microplastic Recognition by Fluorescent Dyes

Due to their predominantly hydrocarbons-based and hydrophobic nature, plastics tend to exhibit strong interaction with lipophilic fluorescent dyes, which bind more readily to microplastics. However, these dyes present certain limitations, primarily due to their non-specific binding properties. As a result, this approach can confirm the presence of microplastics but cannot differentiate between specific types of microplastics [35]. Moreover, the high fluorescent background inherent in environmental samples often leads to false-positive results during identification [103]. To address these challenges, it is necessary to employ separation and digestion techniques during the pre-treatment process or utilize other strategies to eliminate suspended solids and organic matter that interfere with accurate identification.

The most critical aspect of fluorescence-based microplastic identification lies in the selection of appropriate fluorescent dyes and suitable staining protocols. These factors are essential for tracking microplastics in environmental samples [104]. Notably, certain dyes exhibit substantial variation in their excitation and emission wavelengths depending on the solvent used. The Table 3 provides an overview of fluorescent dyes commonly employed for microplastic detection, with partial citations from Silvia Morgana et al. [105].

Nile Red is the most commonly used fluorescent dye for environmental detection of microplastics. Gabriel Erni-Cassola et al. [130] proposed a rapid screening method based on density extraction and filtration to analyze plastic particles in environmental samples, using Nile Red for selective fluorescent staining. Prata J C et al. [131] utilized Nile Red’s strong adsorption to plastic surfaces, where the dye emits fluorescence upon blue light excitation. Image analysis techniques, using an orange filter, allow for the identification and quantification of fluorescent particulate matter. Meyers et al. [132] used red, green, and blue (RGB) data extracted from photographs of Nile Red fluorescently stained microplastics (50–1200 μm) to train and validate the Plastic Detection Model (PDM) and the Polymer Identification Model (PIM), achieving a detection accuracy of 92.8% for the PDM and an identification accuracy of 80% for the PIM during testing.

Rhodamine B is effective for staining PVC and PMMA. Pham Le Quoc et al. [133] successfully used Rhodamine B to stain 2- and 4-micrometer PVC particles and confirmed their stability in seawater. WanYuan Li et al. [134] employed Rhodamine B encapsulated PMMA micro/nanoparticles, enhancing visual clarity for detection. Matteo Cingolani et al. [135] used poorly emissive hyaluronan functionalized with rhodamine B (HA-RB) to stain MNPs, enabling Identification through fluorescence lifetime analysis.

Safranine T exhibits a strong staining effect on various plastics, though its environmental impact limits its application [136]. Lulu Lv et al. [119] compared the staining efficiency of NR, FITC, and ST on PE, PVC, PET, and PS, finding that ST Nile Red was more effective for most microplastics, with FITC showing superior performance on PVC.

Eosin B is effective for identifying PP and PE. K Chouchene et al. [122] utilized EB to identify PP and PE microplastics in the port of Sidi Mansour in southeastern Tunisia and characterize foam surfaces and pipes.

Rhodamine 6G is primarily applied for staining HDPE. In a review of titanium dioxide photocatalysts. Seema Singh et al. [137] noted its use for dyeing HDPE. While less commonly used, other dyes include phenol, Methylene Blue (MB), Methyl Orange (MO), Trypan Blue (TB), Victoria Blue R (VBR), reactive bright blue KN-R, Indigo Carmine (IC), 4-dimethylamino-40-nitrosodiphenylethylene (DANS), 4-Chlorophenol (4-CP), Metanil Yellow, Perylene Di Imide (PDI), iDye, and 1-Pyrenylbutyric Acid N-Hydroxysuccinimide Ester (PBN) are also applied for specific types of plastics. [137,138]

### 3.2. Plastic Identification of Fluorescence Lifetimes for Autofluorescence

In contrast to fluorescent dye staining, which lacks the ability to differentiate between plastic polymers, fluorescence lifetime identification offers the potential to distinguish various plastic types. Fluorescence lifetime measurement and imaging are primarily conducted using FLIM, a tool widely employed in the study of protein–protein interactions, cellular signaling, and the differentiation of spectrally overlapping fluorophores [139]. FLIM can also provide quantitative information about electrical signals, ion and oxygen content, and changes in temperature and pH within cells or the surrounding environment [140,141]. Langhals et al. [142] and Gies et al. [143] demonstrated that fluorescence lifetime measurements in the time domain can identify plastics, marking a significant advancement in the use of FLIM for differentiating microplastic types [100]. As a microspectroscopic technique, FLIM capitalizes on the temporal resolution of fluorescence [144], enabling not only the detection of plastics but also the identification of plastics based on phase-dependent and modulation-dependent fluorescence lifetimes (τ) [145]. The variation in fluorescence lifetimes is directly influenced by parameters such as temperature, chemical additives, aging, and weathering, making it a powerful tool for more precise characterization of plastic types [100].

Despite its advantages, the data obtained from FLIM measurements are raw and necessitate processing through physical and mathematical models. The fitting of FLIM data requires the application of empirical fluorescence lifetime principles and mathematical models, typically involving nonlinear fitting techniques. Inappropriate fitting can introduce uncertainties in fluorescence lifetime calculations, which is particularly problematic when multiple fluorescence lifetimes are involved [146,147]. These uncertainties can negatively impact the reproducibility of results, hinder the accurate differentiation of plastic types, and potentially distort the measurement of plastic fluorescence lifetimes [146].

#### 3.2.1. Identification of Microplastics with Fluorescence Lifetime

Fluorescence experiments involving plastics often yield multiple lifetime components, which are derived from different molecular types or different conformations of the same molecule [148]. These fluorescence lifetimes can be influenced by intermolecular interactions [149]. The fluorescence decay process can be measured using various techniques, including time-dependent single-photon techniques [150], frequency-domain methods, and time-sampling methods [151]. In synthetic plastic polymers, the decay process is primarily mono-exponential, with polymers exhibiting characteristic mono-exponential autofluorescence lifetimes. However, a bi-exponential component may also be present, providing an additional parameter for the advanced classification of plastics [142]. The bi-exponential components influence the mono-exponential decay’s fluorescence lifetime, acting as an average of the two constituents and reflecting their relative contributions to the overall fluorescence signal. Fluorescence lifetimes of materials can be derived using linear fitting techniques and vector analysis. The latter approach offers distinct advantages, providing fit-free data within an interactive two-dimensional vector space, which allows for more accurate and rapid calculations than conventional fitting methods [152]. Substances with distinct fluorescence lifetimes generate separate clusters on the phase-volume diagram, thereby producing phase-volume fingerprints that can be used for material identification.

To facilitate the differentiation and characterization of various types of plastics, Table 4 presents data derived from FLIM measurements of fluorescence lifetimes for various microplastics:

Regarding the impact of heat treatment on the average fluorescence lifetime, it can be inferred that thermal exposure induces alterations in the molecular structure and surface chemistry of microplastic particles, which consequently modulate their fluorescence decay dynamics. Heat treatment may cause polymer chain scission, cross-linking, or oxidation, thereby affecting non-radiative relaxation pathways and resulting in variations in the observed fluorescence lifetime [157]. For example, plastics such as PLA and PA6 exhibit a decrease in lifetime post-heating, due to molecular degradation leading to shortened excited-state lifetimes. Conversely, PPE shows an increase in fluorescence lifetime after heat treatment, potentially reflecting the formation of new fluorescent species or reduced quenching effects [100]. Additionally, thermal processing may modify surface adsorbates, such as impurities or moisture, further influencing fluorescence characteristics [158]. Monitoring these lifetime changes provides an indirect yet insightful indicator of the thermal aging and chemical modifications of microplastics. This understanding is vital for elucidating degradation mechanisms in environmental contexts and enhances the specificity of fluorescence-based detection approaches. Integrating these observations with multi-wavelength excitation and phasor analysis methods offers promising avenues for more accurate classification and identification of heat-affected microplastic particles.

Zhou et al. [75] demonstrated an innovative approach to identifying and differentiating microplastic particles using FLIM phase analysis, combined with a ‘microplastic phase fingerprinting’ database. This technique utilizes autofluorescence excitation at two specific wavelengths (405 nm and 440 nm) for four plastic types—ABS, PET, PVC, and PLA—without requiring additional staining or processing. The study confirmed that microplastic particles can be accurately recognized and classified based on FLIM-phase data, without the need for fitting raw autofluorescence lifetime data.

It was found that the 405 nm laser is effective for short-wave excitation, though it lacks the short pulse widths and high repetition rates required for precise fluorescence lifetime measurements. In contrast, the 440 nm laser, within the white-light laser range, was shown to effectively excite a broader range of autofluorescence signals. These findings align with those of Maximilian Wohlschläger et al. [159], who also noted potential interference from biofilms in the environmental samples, which may alter the fluorescence lifetime of plastic particles. Wohlschläger suggested that constructing a comprehensive FLIM database could mitigate background fluorescence interference caused by biofilms.

Another key aspect of autofluorescence is its decay rate, which varies with excitation wavelength and is a critical parameter for material characterization. Aigars Piruska et al. [160] demonstrated that the autofluorescence of plastic materials exhibits a fast exponential decay, with intensity approaching a limiting value after approximately 300 s of excitation. The decay rate was found to increase with longer laser excitation wavelengths. The study suggests that understanding this feature can aid in reducing background noise and optimizing excitation wavelength selection for plastic detection.

In practical applications, portable fluorescence lifetime analyzers are now available, bridging the gap between theoretical research and field-based microplastic detection. Siyao Xiao et al. [154] developed a compact fluorescence lifetime analysis system for use in water that is capable of detecting both fluorescently labeled and unmodified polystyrene particles. This system could detect microplastics within a size range of 35~140 nm and can detect single polymers at concentrations as low as 0.01 mg/mL, eliminating the need for a fluorescence microscope. Its compact design and reduced production costs make it a practical tool for field-based microplastic detection.

Overall, FLIM technology offers several advantages for identifying and analyzing microplastics. It facilitates the precise and rapid differentiation of various plastic types, accommodates a wide range of materials under varying experimental conditions, and has the potential for broader application through the development of portable systems. To enhance reproducibility across different laboratories and instrumentation platforms, standardized protocols and calibration strategies have been proposed. These include the implementation of reference dyes (e.g., fluorescein, Rhodamine 6G), calibration of instrumental response functions (IRF), and consistent acquisition parameters. In this context, the phasor approach has emerged as a powerful analytical method, offering a fit-free alternative that effectively reduces fitting bias [146]. Moreover, due to its standardized graphical representation, it facilitates robust cross-platform comparisons of fluorescence lifetime data [161], thereby improving the comparability of results obtained from different imaging systems. Complementing these methodological advancements, the ISO 21073:2019 standard [162] provides detailed guidance on fluorescence instrument calibration, laying a foundational framework for achieving reliable and reproducible measurements in the characterization of plastic fluorescence lifetimes. As FLIM technology advances, its role in environmental monitoring and microplastic detection is expected to expand, offering valuable insights for both research and practical applications.

#### 3.2.2. Identification of Larger Plastics by Fluorescence Technology

To differentiate macrolitter from microplastics, it is important to note that while their fundamental chemical compositions are similar [163], the degradation of macrolitter into microplastics often involves the release of various additives, macrolitter leading to alterations in the chemical structure of the plastic [164]. Unlike microplastics, the size of the macrolitter significantly influences fluorescence detection. Medium and large plastics exhibit distinct fluorescence characteristics due to their larger dimensions and more complex structural compositions. For example, variations in the additives and physical properties of these plastics can substantially influence their fluorescence emission profiles [165]. Consequently, the identification and analysis of macrolitter require methodologies that differ considerably from those employed for microplastics.

A notable study by M.F. Sonnenschein et al. [166] on PET demonstrated that variations in composition, additive content, and even color could result in inconsiderable shifts in fluorescence intensity and emission wavelength. These shifts underscore the necessity for tailored fluorescence detection methods for different types of plastics, as the fluorescence response of microplastics is subject to several influencing factors. Table 5 illustrates these shifts in fluorescence behavior for various PET materials based on Sonnenschein and Roland’s analysis:

The data in Table 5 demonstrate significant variability in fluorescence behavior across PET materials. Fluorescence measurements were performed on rectangular blocks (approximately 2 × 3 cm) of PET bottle walls using the RF-6000 spectrophotometer system, Shimadzu, Kyoto, Japan. These results emphasize the challenges of identifying large plastic fragments using fluorescence techniques, as the presence of colorants, flame retardants, and other additives can significantly alter fluorescence characteristics.

Shi et al. [167] conducted a comprehensive literature review on plastics, highlighting a notable knowledge gap between marcoplastics and MNPs. Their review concluded that medium-sized plastics, ranging from 5 mm to 25 cm, play a crucial role in bridging this gap. These medium-sized plastic fragments share commonalities with both microplastics and large plastic fragments, making them essential for understanding the full spectrum of plastic contamination in marine environments.

The fluorescence-based identification of large plastics has been a long-standing laboratory practice. Heinz Langhals [142] demonstrated that many plastics predominantly exhibit mono-exponential fluorescence decay. However, the application of bi-exponential data processing allows for a two-dimensional characterization of polymer structures, even for materials that display similar mono-exponential fluorescence lifetimes. This approach significantly improves the differentiation of chemical compositions in plastic materials, enhancing the sorting efficiency of different plastic types. Table 6 presents fluorescence lifetime measurements of various large plastics.

Wohlschläger et al. [168] utilized light-emitting diodes to excite plastics and measure fluorescence intensity, which was then used to calculate quantum efficiency and signal loss along the optical path. This method facilitated the differentiation of three distinct plastic polymers (PA, PET, and PE), enhancing the precision of plastic-type identification. In a subsequent study, Maximilian Wohlschläger et al. [169] further developed this approach to simulate marine conditions for the detection and identification of PA, PE, and PP plastics in aquatic environments. By incorporating water-specific light loss coefficients, they improved the accuracy of plastic detection and the characterization of different polymer layers under realistic aquatic conditions.

Detecting macrolitter in marine environments is challenging due to the numerous environmental factors that impact plastic degradation. Prolonged exposure to UV radiation, hydrolysis, and oxidizing agents can degrade polymer backbones [170], reducing the tensile properties and promoting the development of surface cracks [171]. These weathering processes complicate the identification of plastic, as fluorescence behavior can be altered over time. For instance, Shi et al. [167] observed that plastic fragments found on a beach exhibit varying weathering characteristics on different sides due to differential exposure to UV radiation and environmental conditions. This finding highlights the complexity of plastic degradation in marine ecosystems, further complicating detection and characterization efforts.

Kenneth M. White et al. [172] identified that during the initial stages of photo-driven degradation of PET films under varying solar radiation and temperature photo-oxidation products exhibited strong fluorescence. Similarly, Mathew Philip et al. [173] examined the weathering of plastic solid waste under both artificial and natural conditions. Their study, which included 1000 and 5000 h of UV exposure, demonstrated that molecular absorption by chromophores, such as carbonyl groups, led to chain scission, creating free radicals that could react with diffused oxygen or water molecules. This degradation process ultimately resulted in surface discoloration and gloss loss, along with the development of microcracks, and the formation of by-products that significantly impacted the fluorescence lifetime measurements of the plastics. These findings provide a crucial theoretical foundation for the practical application of fluorescence-based plastic detection.

Beyond intrinsic photochemical changes, an important yet underexplored dimension is the potential interaction between fluorescent dyes and plastic weathering by-products. While FLIM-based techniques frequently rely on external staining agents—such as Nile Red, BODIPY, or other solvatochromic dyes—to enhance signal contrast, the local chemical environment generated by polymer degradation can directly affect dye behavior. Weathering by-products, particularly those formed during photo-oxidation (e.g., carboxylic acids, ketones, aldehydes, peroxides), may alter surface polarity, hydrogen bonding capacity, and microviscosity, all of which can influence dye binding, fluorescence intensity, spectral characteristics, and, critically, fluorescence lifetime.

Recent studies have begun to investigate these complex interactions. For instance, Wakaba Idehara et al. [174] explored the performance of Nile Red staining for microplastics subjected to controlled surface oxidation and found that oxidative weathering directly altered the staining behavior and fluorescence characteristics of the polymer surfaces. Specifically, oxidized plastics—such as UV-aged polyethylene—exhibited diminished fluorescence signal intensity and shifts in emission profiles compared to their non-weathered counterparts. These effects were attributed to changes in surface polarity and the formation of oxygen-containing functional groups, which influenced Nile Red’s partitioning behavior, photostability, and local microenvironment.

This evidence suggests that dyes commonly used in microplastic detection are not chemically inert with respect to weathered polymer matrices. Instead, the oxidative degradation products—such as carbonyls, hydroxyls, and peroxides—can modulate dye binding, local solvation, and fluorescence lifetimes through altered surface energetics, hydrogen bonding, or even quenching mechanisms [175]. In a related study, Than Htun et al. [176] examined the fluorescence emission of HDPE and LDPE and observed that prolonged UV exposure led to decreased fluorescence intensity and shortened fluorescence lifetimes, as a result of photo-oxidative degradation. This underscores the direction correlation between the weathering characteristics of plastics and their fluorescence lifetimes. Such physicochemical interactions can introduce variability in fluorescence measurements, particularly in FLIM applications where lifetime contrast is key for material differentiation [177].

Moreover, while the phasor approach offers a robust, model-free method to visualize lifetime distributions [146], its sensitivity to environmental conditions necessitates an understanding of how degradation-induced changes affect lifetime contrast. This underlines the need for careful calibration and potentially the development of new protocols or reference materials for aged plastics.

In addressing the complexities of detecting plastic in aquatic environments, Jiajun Duan [178] investigated the interactions between microplastics and aquatic ecosystems. The study concluded that photodegradation was the primary mechanism driving plastic weathering, while microbial degradation and biological ingestion were key contributors to plastic breakdown in the ocean. However, detection is further complicated by the background fluorescence from colored dissolved organic matter (CDOM), phytoplankton, and other pollutants in the marine environment. Additionally, environmental factors such as salinity, turbidity, and the presence of organic materials further affect the fluorescence signals of plastics. The high salinity and turbidity in marine areas can alter light absorption and scattering properties, which may impact the intensity, clarity, and accuracy of fluorescence signals [179]. Organic matter in the water, including dissolved organic carbon (DOC) and humic substances, can contribute additional fluorescence that interferes with the self-fluorescence of plastics [180]. Moreover, biological organisms such as phytoplankton, zooplankton, and biofilms can also emit fluorescence, making it difficult to distinguish between environmental fluorescence and plastic autofluorescence [181]. These complex environmental interactions highlight the need for advanced detection methods capable of minimizing or compensating for such interferences in fluorescence-based plastic identification.

Jumar Cadondon et al. [182] addressed the challenge by utilizing 405 nm lidar-generated fluorescence spectra to detect submerged plastics. Their experiments revealed that submerged plastics, such as PS, PP, PET, and HDPE, exhibited fluorescence intensities approximately twice as high when submerged compared to their dry state. Although this approach was effective in freshwater and pond surface waters, high signal-to-noise ratios in ocean environments and the impact of plastic weathering, presented challenges for accurate plastic identification in seawater. Nevertheless, the study demonstrated that water refraction and absorption did not significantly alter the fluorescence spectrum, confirming the feasibility of fluorescence-based detection in marine environments. Based on these findings, this paper proposes monitoring changes in plastics’ autofluorescence intensity and lifetime to facilitate the differentiation of plastic types and to separate them from oceanic fluorescence backgrounds. Establishing a comprehensive fluorescence database for marine plastics could further enhance detection accuracy. Moreover, fluorescence intensity and lifetime could serve as valuable indicators for assessing the extent of plastic weathering and degradation [183].

In this paper we review the advancements in the application of fluorescence lifetime measurements, and this paper proposes an innovative hypothesis: by analyzing both the fluorescence intensity and fluorescence lifetimes of plastics, it is possible to more effectively identify different plastic types in oceanic environments and gain insights into their specific types and weathering patterns. In this paper, we aim to advance the application of fluorescence lifetime measurements by systematically investigating the variations in fluorescence lifetimes and decay dynamics of plastics subjected to oceanic weathering conditions. While the utility of fluorescence lifetime analysis for characterizing plastic materials is well established, further work is needed to contextualize these measurements within the complex and variable conditions of natural aquatic environments. By correlating lifetime changes with environmental degradation factors such as UV exposure, salinity, and biofouling, this approach seeks to enhance the environmental relevance of fluorescence-based plastic characterization and support more accurate interpretation of in situ degradation processes.

In addition to reviewing the current advancements in marine plastic identification using fluorescence technology, this paper explores future directions for improving plastic detection in aquatic environments. Through this examination, the research aims to establish a scientific basis for the efficient identification and recycling of marine plastics, ultimately contributing to more effective strategies for the management and reduction in marine plastic pollution.

## 4. Prospects

Given the unique advantages of fluorescence spectroscopy for plastic identification, advancing research on its application is essential. Future research should undertake a comprehensive investigation into the autofluorescence properties of various plastics, focusing on key parameters such as fluorescence intensity and fluorescence lifetime. This foundation will enable the refined classification and identification of plastic types. In addition, technological innovations are critical, for example, the integration of fluorescence lifetime imaging with other experimental and analytical techniques holds promise for the rapid and precise classification and identification of plastic types.

Developing equipment and methodologies tailored to diverse environmental conditions is another priority. Specifically designing solutions for plastic identification in complex environments, such as those with high background fluorescence or environmental contaminants, poses a significant challenge. For fluorescent dye-labeled plastic identification, major obstacles in fluorescent dye-labeled plastic identification include the non-specificity of fluorescent dyes and interference from high environmental background fluorescence. Future research should prioritize enhancing the specificity of fluorescent dyes and devising strategies to suppress or eliminate such environmental interference.

For fluorescence lifetime-based plastic identification, autofluorescence excitation not only enables plastic identification but also reveals the weathering and degradation processes affecting the plastic. This capability supports more effective classification and processing of plastics. In this paper, we propose that by subjecting plastics to controlled environmental stressors—such as UV irradiation, salinity, and temperature—it is possible to induce measurable changes in fluorescence lifetime and intensity that reflect the degree of weathering. By systematically correlating these fluorescence parameters with known weathering durations and conditions, researchers can simulate marine degradation processes in laboratory settings and gain insights into the transformation pathways from macroplastics to microplastics. Moreover, to enhance the practical relevance of fluorescence-based plastic identification, future research should explore its application in real-world marine ecosystems. For instance, investigating how fluorescence techniques perform in detecting plastics within living marine organisms [184] —such as zebrafish [185], blue mussels [186], and jellyfish [187] can provide valuable insights into bioaccumulation, biodistribution, and trophic transfer of plastic particles [188]. The integration of fluorescence imaging and staining in in vivo or in situ models [189] would help bridge the gap between laboratory analyses and environmental realities, ultimately supporting improved recycling strategies for marine plastics and contributing to the protection of aquatic ecosystems.

The research and application of fluorescence technology for plastic identification offers considerable potential to support the recycling and reuse of plastic waste. Effective categorization of plastic waste is a foundational step toward advancing a circular economy, reducing plastic pollution, and protecting the environment. Consequently, the use of fluorescence technology in plastic identification holds substantial social and environmental significance.

This review paper highlights the potential of fluorescence technology for rapid plastic detection and environmental assessment through a comprehensive analysis of its application in plastic identification. Nevertheless, several challenges remain that limit their broader application in real-world environments. For instance, photobleaching and fluorescence quenching may significantly reduce signal stability and detection sensitivity, particularly in complex or heterogeneous environmental matrices. Additionally, the lack of standardized protocols for fluorescence-based detection hinders reproducibility and comparability across different studies and laboratories. Potential environmental risks related to the toxicity and leaching of fluorescent tracers—especially under in vivo and in situ conditions—also require careful consideration. Therefore, future research should aim to enhance detection efficiency and throughput while simultaneously addressing these technical and ecological limitations. Moreover, expanding the use of fluorescence technologies in plastic recycling and long-term ecological monitoring represents a promising direction for advancing sustainable solutions in environmental science.

In parallel with advances in fluorescence instrumentation and environmental validation, the integration of machine learning (ML) algorithms and image-processing techniques has emerged as a promising direction for enhancing the classification and identification of plastic types from fluorescence data. Recent studies have demonstrated that convolutional neural networks (CNNs) and other supervised learning frameworks can effectively extract morphological, spectral, and textural features from fluorescence-stained microplastic images, significantly improving classification accuracy under complex conditions [132]. In particular, the use of annotated datasets derived from Nile Red-stained particles or fluorescence lifetime imaging (FLIM) has enabled automated polymer type differentiation, reducing dependency on manual interpretation and subjective bias. Moreover, fit-free phasor analysis combined with unsupervised clustering techniques has shown potential in separating polymers based on lifetime distributions without requiring extensive model fitting [152]. To advance these computational methodologies toward real-world applicability, future efforts should focus on developing standardized and diverse fluorescence image datasets, constructing domain-adaptive models that are robust to environmental variability, and designing real-time image analysis pipelines suitable for in situ deployment. The integration of these data-driven frameworks with fluorescence detection technologies will be instrumental in facilitating large-scale, automated plastic monitoring for both ecological assessment and waste management applications.

## Figures and Tables

**Table 1:** Comparison of fluorescence-based imaging techniques for plastic identification.

Technique	Resolution	Detection Limit	Throughput	Key Advantages	Limitations	Reference
Confocal Laser Scanning Microscopy (CLSM)	Lateral: ~200–250 nm Axial: ~500–700 nm	≥1 μm	Low	High spatial resolution; optical sectioning; 3D imaging	Photobleaching; limited depth; slow scanning maintenance	[74,76]
Fluorescence Lifetime Imaging Microscopy (FLIM)	Depends on platform (typically ~300 nm)	~0.5–2 μm (lifetime contrast)	Medium	Quantitative contrast independent of intensity; detects environmental effects	Complex setup; long acquisition time	[75,77]
Widefield Fluorescence Microscopy	~250–300 nm	≥1 μm	High	Fast imaging; simple setup	Poor axial resolution; high background noise	[76]
Imaging Flow Cytometry	~500–1000 nm	~2–20 μm	Very High	High-throughput analysis of particles in flow; Acquisition of fluorescence and structural data	Requires suspended particles; lower spatial resolution	[78,79]
Two-Photon Microscopy	~300–500 nm lateral	≥0.5 μm	Low	Deep penetration; minimal photodamage; suitable for in vivo imaging	High cost; slow scanning; needs pulsed laser	[80,81]

**Table 2:** Different parameters for heat treatment of plastics.

Plastic Type	Temperature (°C)	Heat Treatment Time (h)	Fluorescence Lifetime Change	Fluorescence Intensity Changes
Acrylonitrile-butadiene-styrene copolymer (ABS)	140	12	−0.178	Slightly enhanced
Poly(p-phenylene oxide (PPO)	160	12	−2.618	Significantly enhanced
Polyamide 6 (PA)	160	12		Higher photon yield
Polyethylene terephthalate (PET)	210~220	12	−0.02	Slightly enhanced
Polylactide (PLA)	140	12		
Polyurethane (PU)	160	12		

**Table 3:** Fluorescent dyes related research data.

Dyes	$\lambda_{ex}\backslash \lambda_{em}$	Target Polymers	Advantage	Limitation	Reference
Nile Red	In nonpolar lipids: 460 nm/620 nm In polar lipids: 543 nm/620 nm 560 nm/635 nm	PE, PP, PVC, EVA, PVA, PTFE, PET, PS, PA, acrylic, PU	- Broad compatibility with various plastic types—works efficiently on a wide range of environmental samples. - High adsorption and fluorescence intensity. - Non-toxic at use concentrations. - Solvent-discolor is easy to visualize and analyze.	- Weak signal with PA, PVC, and polyester. - Poor dyeing performance on fibers. - Non-specific binding to natural organic matter may cause false positives.	[104,106,107,108,109,110,111,112]
Rhodamine B	In ethanol: 540 nm/565 nm In methanol: 556 nm/580 nm	PE, PP, PU, PVC, PMMA	- Good sensitivity to a variety of plastic polymers—high fluorescence emissivity and good stability under different pH conditions. - Effectively stains different types of polymers. - High affinity for more hydrophilic structures.	- Poor effectiveness with PS particles. - Highly toxic at low concentrations. - Small size: Only partially stained. - Inability to effectively differentiate between certain biodegradable plastics and organic biomaterials. - Not yet applied to environmental samples.	[113,114,115,116]
Safranine T	In water: 520 nm/563 nm	PE, PP, PU, PVC	- Good sensitivity to common plastics—high fluorescence emissivity—excellent stability over different pH conditions. - Effectively stains a variety of polymer types.	- Documented harmful effects on biological systems—not suitable for hydrophobic plastic materials. - Not yet applied to environmental samples.	[117,118,119,120]
Eosin B	In water: 521 nm/544 nm In ethanol: 527 nm/550 nm	PE, PP	- Good staining results for PE and PP plastics—common dyes used for fluorescence detection. - Effective for small particles (up to 0.1 mm in size).	- Easily stains natural fibers and misclassifies. - Limited environmental sample testing (only one study available).	[121,122]
Rhodamine 6G	In polar lipids: 527 nm/555 nm	HDPE	- Extremely high photostability—high quantum yield. - Low cost. - Near-maximum absorption. - Frequently used as a tracking dye to monitor water flow rate, direction, and transmission - pH insensitive with high absorption coefficients.	- Quenching effect on some metal ions (e.g., copper Cu^2+^). - Difficult to distinguish between biomaterials and plastics. - Toxic and hazardous in direct contact with skin or if inhaled.	[123,124,125,126]
Fluorescein Isothiocyanate (FITC)	In polar lipids: 488 nm/517 nm	PS, PVC, PE, PET	- Good staining results for PS, PVC, PE, PET—high emissivity and sensitivity. - High-contrast visualization: provides excellent microscopic imaging. - Simple operation: can be directly covalently bonded to other molecules.	- High rate of photobleaching: Requires caution during use. - Sensitive to pH: This may affect its utility under certain conditions. - Difficulty in distinguishing between plastics and biomaterials	[127,128,129]

**Table 4:** FLIM measurements of fluorescence lifetime for various microplastics.

Author	Band	Plastic-Type	Status	Average Fluorescence Lifetime (ns)	Experimental Equipment
Adrian Monteleone et al., 2021 [100]	470 nm 440 nm	PLA	* DIN Heating 12 h	2.864 (±0.035)	Fluorescence Lifetime Imaging Microscopy (FLIM) System A modular Leica TCS SP8 FALCON (FAst Lifetime CONtrast) system (Leica Microsystems GmbH, Wetzlar, Germany) equipped with an HC PL APO 20×/0.75 Dry CS2 objective lens was employed for fluorescence lifetime imaging of microplastic particles. Image resolution: 512 × 512
		PPE	* DIN Ambient/Heating 12 h	8.143 (±0.060)	
		PA6	* DIN Heating12 h	4.529 (±0.008)	
		ABS	* DIN Ambient/Heating 12 h	3.850 (±0.033)	
		PU	* DIN Ambient	4.224 (±0.010)	
		PET	* DIN Ambient/** ASTM Ambient	3.519 (±0.090)/3.564 (±0.126)	
M Wohlschläger et al., 2024 [153]	488 nm	HDPE	Optical LP Filters Single Material FD-FLIM	1.68 (±0.07)	A frequency-domain fluorescence lifetime imaging (FD-FLIM) camera system, model pco.flim from Excelitas PCO GmbH, Kelheim, Germany Image resolution: 1008 × 1008 pixels
			Optical BP Filter Single Material FD-FLIM	3.52 (±0.21)	
			Optical BP Filter multi-material FD-FLIM	3.24 (±0.60)	
Siyao Xiao et al., 2024 [154]	445 nm	PS	COOH-PS	3.52 (±0.23)	Fluorescence Lifetime Analysis System (FLA Kit) Developer: FLIM LABS (Rome, Italy) The system has a limit measurement of 0.01 mg/mL
			NH2-PS	1.98 (±0.07)	
			Micro PS	2.28 (±0.12)	
			Nano PS	2.39 (±0.16)	

* DIN [155] (Deutsches Institut für Normung) is Germany’s national standardization body. German standards have strict testing and certification requirements for plastic materials’ mechanical, thermal, and chemical resistance. ** ASTM [156] (American Society for Testing and Materials) publishes many standards on plastics and their products, and the American standards are also very detailed on the performance testing of plastics, covering standardized test methods for all aspects, from raw materials to finished products.

**Table 5:** Table of wavelength shifts in fluorescence intensity between different PETs.

Component	Color	Fluorescence Intensity	Excitation Wavelength (nm)	Fluorescence Wavelength (nm)
Lemonade PET bottle	Blue	500	380	495
		355	460	495
Water (PET 100%, 500 mL)	Bright Blue	762	335	435
Water (PET 25%, 600 mL)	Bright Blue	1385	345	415
Citrus Lemonade Bottle (1500 mL)	Blue	3732	370	445

**Table 6:** FLIM measurements of fluorescence lifetime of large plastics.

Author	Band	Plastic-Type	Status	Average Fluorescence Lifetime (ns)	Installations
Heinz Langhals et al., 2015 [142]	403 nm	PMMA		0.841	Fluorescence lifetime measurement equipment: PicoQuant (Berlin, Germany) FluoTime 300; Pico Quant PicoHarp 300 (PC-405 laser; 403 nm).
		PS		3.290	
		PC		1.038	
		PET	Soft Drink Bottles	1.840	
			Plates	4.466	
		PE	LDPE	2.19	
			HDPE	<0.2	
			EHDPE	1.58	
		Silicone	Binder Sn	3.078	
			Binder Pt	3.162	
			Binder Pt (50)	3.114	
			Hose	4.333	
		Delrin^®^ (POM)	DuPont’s polyformaldehyde	4.024	
		Luran^®^ (ASA)	Styrene-polyacrylonitrile copolymer from BASF	3.976	
		Ultramid^®^ (PA)	Polyamide with glass fibers from BASF	3.784	
